# Nutritional Influences on the Brain in ADHD: Evidence from Neuroimaging Studies

**DOI:** 10.3390/neurolint18060107

**Published:** 2026-05-29

**Authors:** Daniele Corbo, Roberto Gasparotti, Francesca Bozzetti, Stefano Renzetti, Laura Clara Grandi, Antonio Vita, Giacomo Deste

**Affiliations:** 1 Department of Medical and Surgical Specialties, Radiological Sciences and Public Health, University of Brescia, 25123 Brescia, Italy; 2 Neuroradiology Unit, ASST Spedali Civili of Brescia, 25123 Brescia, Italy; 3 Neuroradiology Unit, Diagnostic Department, University Hospital of Parma, 43121 Parma, Italy; francesca.bozzetti@unipr.it; 4 Department of Medicine and Surgery, University of Parma, 43121 Parma, Italy; 5 Department of Experimental and Clinical Medicine, University of Florence, 50134 Florence, Italy; laura.grandi@unimib.it; 6 Department of Biotechnology and Biosciences and NeuroMI (Milan Center for Neuroscience), University of Milano-Bicocca, 20126 Milano, Italy; 7 Department of Mental Health and Addiction Services, ASST Spedali Civili of Brescia, 25123 Brescia, Italy; 8 Department of Clinical and Experimental Sciences, University of Brescia, 25123 Brescia, Italy; 9 Department of Mental Health and Addiction Services, ASST Valcamonica, 25040 Esine, Italy

**Keywords:** nutrition, neuroimaging, MRI, ADHD, cognition

## Abstract

Background/Objectives: Attention-deficit/hyperactivity disorder (ADHD) is increasingly recognized as a neurodevelopmental condition shaped by early-life biological and environmental factors. Emerging evidence highlights the role of nutrition in modulating key brain processes involved in ADHD, from gestational development through childhood. This review aims to examine how dietary interventions influence neuroimaging outcomes in individuals with ADHD, assessing whether nutritional approaches can modulate brain structure, function, or connectivity. Methods: A systematic search of PubMed, Scopus, and Web of Science was conducted to identify studies examining the effects of dietary interventions on neuroimaging outcomes in individuals with ADHD. Study quality was assessed using Cochrane RoB 2.0, ROBINS-I, the Newcastle–Ottawa Scale, and the JBI Critical Appraisal Checklist, according to study design. Results: A total of 1059 records were identified, and 4 studies met the final inclusion criteria. The included studies suggest that prenatal vitamin D exposure, omega-3 fatty acids, and micronutrients such as zinc may be associated with structural, functional, and neurometabolic brain characteristics relevant to ADHD. Reported findings included associations with brain volume, glutamatergic regulation, white matter organization, resting-state network integrity, and inattentive symptom. Conclusions: Current evidence supports the hypothesis that nutrition may influence neurodevelopmental processes involved in ADHD, including brain maturation and neural network organization. Although findings remain heterogeneous and limited in number, nutrition appears to represent a biologically plausible and potentially modifiable factor within the developmental framework of ADHD. Further longitudinal and multimodal neuroimaging studies are needed to clarify the mechanisms linking nutrition, brain development, and ADHD.

## 1. Introduction

Dietary interventions can profoundly shape the composition and activity of the gut microbiota, which in turn influences the host’s ability to extract nutrients and bioactive molecules such as neurometabolites, vitamins, and short-chain fatty acids [1]. Many of these compounds, including serotonin and gamma-aminobutyric acid (GABA), exert neuroactive effects by modulating neural signaling within the enteric nervous system and ultimately affecting brain function and behavior [2]. This bidirectional gut–brain axis integrates neural, endocrine, and immune pathways to maintain homeostasis, providing a mechanism through which the gastrointestinal system can influence central nervous system activity [3]. Hence, nutritional approaches, based on prebiotics and broader dietary strategies, are increasingly viewed as promising candidates for long-term, sustainable support of mental health, given their potential to reshape gut microbial activity and associated neurobiological pathways. The effects of specific dietary interventions, such as micronutrient supplementation, elimination diets, and assessments of nutritional status, have been documented across several neuropsychiatric and metabolic conditions. In the case of Attention-Deficit/Hyperactivity Disorder (ADHD), however, the extent to which dietary modifications can meaningfully improve symptomatology remains a matter of ongoing debate. Although various nutritional approaches have shown promising results in some individuals, the robustness, generalizability, and underlying mechanisms of these effects are still not fully understood. ADHD is a neurodevelopmental condition marked by symptoms of age-inappropriate inattention, hyperactivity, and impulsivity, and it frequently co-occurs with other psychiatric disorders, including oppositional defiant disorder [4]. Although ADHD has been widely investigated, its origins remain uncertain, with genetic and epigenetic influences, gene–nutrition interactions, environmental exposures, and stress all likely contributing to its development [5]. Optimal maternal nutrition before and during pregnancy is essential for fetal brain development, with growing evidence highlighting the importance of key nutrients such as folic acid and vitamin D [6]. Deficiencies in these and other micronutrients have been linked to long-term impairments in children’s physical and cognitive development, as well as increased vulnerability to mood disorders like anxiety and depression [7]. Moreover, insufficient vitamin availability during critical prenatal and early postnatal windows may elevate the risk of neurodevelopmental conditions, including Autism Spectrum Disorder and ADHD [8]. Multiple neuroimaging studies suggest that ADHD may be associated with a lag in brain maturation. Specifically, children with ADHD often exhibit delayed development in key cortical features—including overall volume, cortical thickness, and surface area—when compared to typically developing controls [9]. These neuroanatomical differences are most evident in regions involved in attention regulation, executive function, and impulse control, and may reflect a slower trajectory of cortical maturation rather than permanent structural deficits [10]. Actually, ADHD treatment predominantly consists of psychoeducation, behavioural therapy and medication [11]. Although pharmacological treatments are widely used, their effects do not last throughout the entire day and they can produce adverse effects such as sleep disturbances, reduced appetite, headaches, and gastrointestinal discomfort, which often lead to treatment discontinuation [12]. These limitations highlight the need for new therapeutic approaches that target the underlying mechanisms contributing to ADHD. Among emerging therapeutic options, dietary interventions are gaining attention for their potential to influence ADHD symptoms by acting on the intricate molecular communication pathways linking the gut microbiota, the gastrointestinal system, and the brain. The aim of this review is to examine how dietary interventions influence neuroimaging outcomes in individuals with ADHD, exploring whether nutritional approaches can modulate brain structure, function, or connectivity in this disorder.

## 2. Materials and Methods

### 2.1. Study Experimental Design

This systematic review was conducted following a re-adaptation of the PRISMA flow [13]. This systematic review is registered in the PROSPERO database with ID number CRD420261364492. The research was carried out in the period between November 2025 and January 2026.

### 2.2. Inclusion Criteria

Eligible studies met the following criteria: peer-reviewed original research or review articles; human populations; assessment of nutritional factors (dietary patterns, nutrients, supplementation, or nutritional biomarkers); use of structural or functional neuroimaging techniques (magnetic resonance imaging (MRI), functional-MRI (fMRI), diffusion tensor imaging (DTI), positron mission imaging (PET)); and inclusion of a clinical diagnosis or validated assessment of mental disorders. Studies published in English without limitation of the period were considered.

### 2.3. Study Selection

A literature search was conducted in PubMed/MEDLINE, Scopus, Google Scholar and Web of Science to identify studies examining the relationship between nutritional factors and neuroimaging findings in ADHD, limited to human studies. The primary PubMed search string was:

(ADHD OR “attention-deficit/hyperactivity disorder”) AND (nutrition OR diet OR “dietary patterns” OR micronutrients OR “omega-3” OR “fatty acids” OR “iron” OR “zinc”) AND (MRI OR “magnetic resonance imaging” OR fMRI OR “functional connectivity” OR DTI OR “diffusion tensor imaging” OR “white matter” OR “gray matter” OR “cortical thickness” OR “brain volume” OR “fractional anisotropy” OR “MRS”) AND (child OR children OR pediatric OR adolescence OR neurodevelopment) AND (humans [MeSH Terms]).

**Figure 1:**
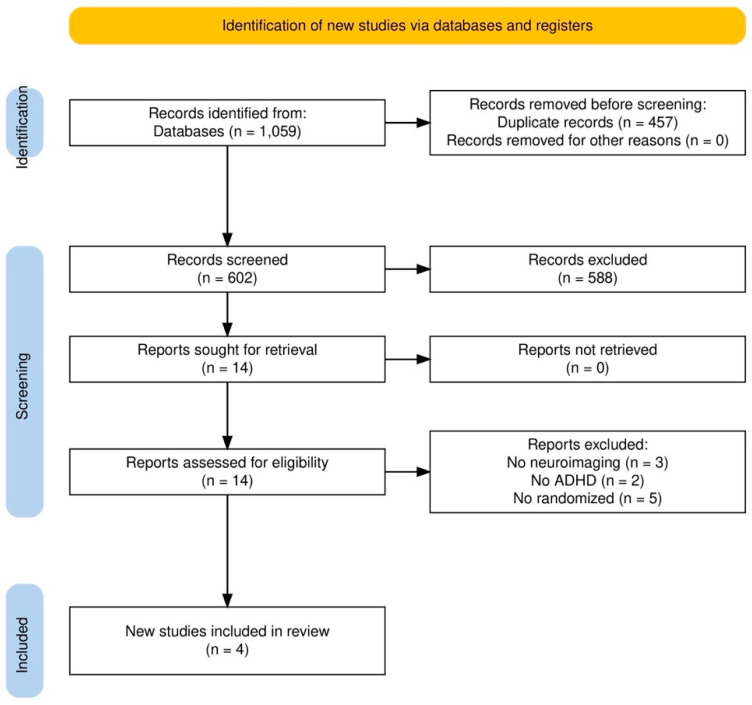
Flowchart of the review process.

Eligible studies were peer-reviewed articles involving children or adolescents with a clinical diagnosis of ADHD, reporting structural or functional neuroimaging outcomes in relation to dietary factors, specific nutrients, or nutritional biomarkers. Studies were excluded if they focused on neurodegenerative diseases, aging populations, or conditions other than ADHD, or if they lacked either neuroimaging data or nutritional measures. In accordance with the preregistered PROSPERO protocol, only randomized studies were included. Two open-label studies initially retained were subsequently excluded to ensure full protocol adherence. Titles and abstracts of all retrieved records were screened, followed by full-text assessment of potentially relevant articles. Data extracted from included studies comprised sample characteristics, diagnostic criteria, nutritional exposure or intervention, neuroimaging modality and analytic approach, and main findings linking nutritional factors to brain structure or function in ADHD. Due to substantial heterogeneity in study designs, nutritional assessment methods, and neuroimaging modalities, a quantitative meta-analysis was not methodologically appropriate. The findings were synthesized using a structured narrative approach within the framework of a systematic review. Two reviewers independently screened titles and abstracts, followed by full-text assessment of potentially relevant articles. Inter-coder agreement was excellent (κ = 0.84), indicating almost perfect agreement between coders. Disagreements were resolved through discussion or consultation with a third reviewer. Database inclusion, exclusion, secondary searches and final inclusion were summarized in the flow diagram (Figure 1).

We found 1059 hits from our initial search, among which, after adjusting for duplicates, 602 articles were retained and screened for title and abstract. Additionally, 588 of these were discarded as not meeting the inclusion criteria. The full texts of the remaining 14 articles were assessed for eligibility and the reference lists of these articles were searched for relevant records, but this search yielded no additional articles. As a last step, the entire manuscript texts of the final full list of 14 included articles were further reassessed to ensure that they meet the inclusion/exclusion criteria and 4 of these were excluded. The total number of articles that met the inclusion criteria was 4. Risk of bias was assessed independently by two reviewers using RoB 2.0 for randomized trials and ROBINS-I for non-randomized studies.

The following information was extracted from each included study: (1) authors and year of publication; (2) sample size; (3) participant characteristics (demographic information of the sample, clinical diagnosis); (4) neuroimaging study characteristics (type of imaging, outcome measure); and (5) main results.

Risk of bias for randomized controlled trials was assessed using the Cochrane Risk of Bias 2.0 tool [14]. Each study was evaluated across five domains: randomization process, deviations from intended interventions, missing outcome data, measurement of outcomes, and selection of reported results. Non-randomized studies (observational and Mendelian randomization) were assessed using the ROBINS-I tool. Two reviewers independently performed all assessments, and disagreements were resolved by consensus. Results are presented in Table 1 and Table 2.

The methodological quality of cohort and case–control studies was assessed using the Newcastle–Ottawa Scale (NOS), which evaluates three domains: Selection, Comparability, and Outcome/Exposure. Two reviewers independently performed the assessment, and discrepancies were resolved by consensus. NOS scores for each study are presented in Table 3.

Analytical cross-sectional studies were assessed using the JBI Critical Appraisal Checklist, which evaluates methodological quality across eight domains, including sampling strategy, measurement validity, identification of confounding factors, and appropriateness of statistical analyses. Two reviewers independently performed the assessment, and disagreements were resolved by consensus. Results are presented in Table 4.

## 3. Results

Among the identified studies, four provided the most direct evidence regarding the relationship between nutritional factors and neuroimaging findings in ADHD and are summarized in Table 5.

The included studies demonstrated substantial methodological heterogeneity in participant characteristics, nutritional exposures, neuroimaging modalities, and analytical approaches. Neuroimaging techniques included structural magnetic resonance imaging (MRI), functional MRI (fMRI), resting-state fMRI, magnetic resonance spectroscopy, and imaging-derived phenotypes derived from large neuroimaging datasets. Nutritional variables investigated across studies included prenatal vitamin D exposure, maternal diet quality, multivitamin supplementation, omega-3 fatty acids, broad-spectrum micronutrient supplementation, and genetically predicted micronutrient concentrations such as zinc, magnesium, and vitamin B12. Study populations also differed substantially with regard to age, clinical characteristics, and methodological design, ranging from randomized placebo-controlled intervention studies in children with ADHD to large population-based cohort investigations and genetically informed causal inference analyses. Outcome measures varied across studies and included structural brain volumes, functional connectivity, neurometabolite concentrations, cognitive-control activation patterns, and imaging-derived phenotypes associated with neurodevelopmental risk.

**Figure 2:**
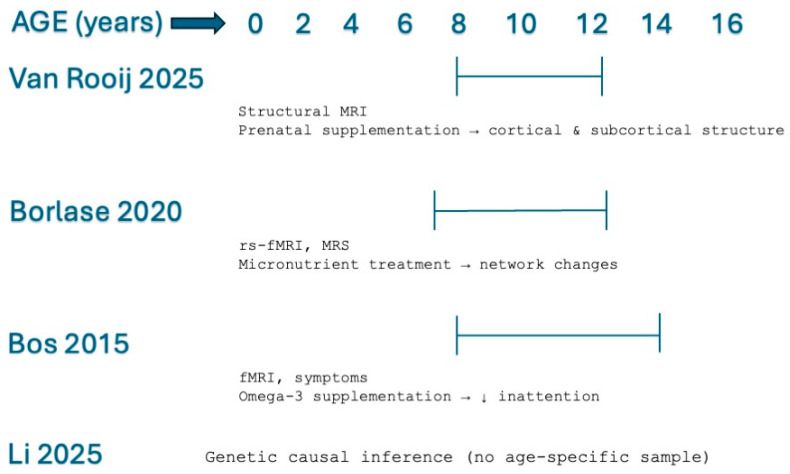
Minimal schematic visualization of age ranges and findings [12,15,16,17].

Figure 2 provides a schematic visualization of the age ranges investigated across studies, together with the associated neuroimaging findings. This diagram highlights the developmental windows covered by each study and facilitates comparison across heterogeneous designs.

Van Rooij et al. [12] investigated associations between prenatal nutritional exposure, childhood brain morphology, and ADHD- and ASD-related traits within the Generation R population-based cohort. Structural MRI analyses were conducted in 3737 children with a mean age of 10.12 years. Maternal vitamin D levels, multivitamin supplementation, folate levels, and overall maternal dietary quality during pregnancy were examined in relation to offspring neurodevelopmental outcomes. The study demonstrated that maternal vitamin D concentrations and better dietary quality were associated with structural variations in childhood brain volume patterns. Independent component analyses identified structural variations involving temporal/parietal, frontal–temporal, subcortical, and hippocampal regions. Specifically, higher maternal vitamin D concentrations were associated with temporal/parietal and frontal–temporal components, whereas better dietary quality was associated with temporal/parietal, frontal–temporal, and subcortical components. Improved maternal dietary quality was significantly associated with lower ADHD trait scores in offspring, whereas multivitamin supplementation showed marginal associations and vitamin D levels were not independently associated with ADHD traits after multivariable adjustment. In parallel, higher maternal vitamin D concentrations and better dietary quality were associated with lower ASD trait scores. Mediation analyses further suggested that selected associations between prenatal dietary variables and ADHD traits were partially mediated through structural brain volume patterns, particularly involving temporal/parietal regions previously implicated in attentional and executive processes. Additional analyses demonstrated that several associations remained relatively stable after adjustment for demographic and pregnancy-related covariates, including socioeconomic variables, maternal smoking, and gestational characteristics. Overall, the findings suggested that prenatal nutritional exposures are associated with neurodevelopmental trajectories relevant to ADHD and ASD, potentially through associations with structural brain development.

Borlase et al. [15] evaluated the effects of broad-spectrum micronutrient supplementation in children with ADHD using multimodal neuroimaging approaches, including structural MRI, resting-state functional MRI, and magnetic resonance spectroscopy. The randomized placebo-controlled trial included 27 male participants with a mean age of 10.75 years. The primary objective was to investigate whether micronutrient supplementation produced measurable changes in neurometabolite levels, structural brain measures, or large-scale functional connectivity after 10 weeks of treatment. No statistically significant differences were observed between treatment and placebo groups in the primary analyses. However, exploratory analyses suggested several potential neurobiological changes associated with micronutrient treatment, including decreased glutamate concentrations in the prefrontal cortex, reduced striatal choline levels, increased grey matter volume in the anterior thalamus, increased white matter within the fornix, and possible alterations in the integrity or connectivity of the default mode, dorsal attention, and frontal executive networks. Additional exploratory findings suggested possible changes involving frontal executive systems and attentional networks implicated in cognitive regulation and inhibitory control. The authors emphasized that these observations emerged from exploratory analyses conducted in a small sample and without correction for multiple statistical comparisons; therefore, the findings require cautious interpretation. Overall, the study provided preliminary evidence suggesting that broad-spectrum micronutrient supplementation may be associated with subtle neurometabolic and connectivity-related changes in neural systems relevant to ADHD symptomatology, although larger adequately powered studies are required to confirm these findings.

Bos et al. [16] investigated the effects of omega-3 polyunsaturated fatty acid supplementation on ADHD symptoms and cognitive-control processes using task-based functional MRI. The study included 40 boys with ADHD and 39 healthy controls in a 16-week double-blind randomized placebo-controlled trial. Participants underwent EPA/DHA supplementation followed by functional neuroimaging assessment during cognitive-control paradigms designed to evaluate attentional and dopaminergic cognitive-control systems. Following supplementation, reductions in parent-rated inattentive symptoms were observed in participants with and without ADHD, suggesting modest behavioral improvements associated with omega-3 fatty acid intake. However, no significant effects of omega-3 supplementation were identified on functional MRI measures of brain activity during cognitive-control tasks. Specifically, the intervention did not significantly alter neural activation during cognitive-control paradigms or dopaminergic cognitive-control systems implicated in ADHD. The authors therefore concluded that the observed behavioral improvements were not accompanied by measurable changes in neural activity detected using the employed fMRI paradigms. These findings suggested that omega-3 supplementation may exert subtle behavioral effects without producing detectable alterations in task-related functional activation, or alternatively that the imaging paradigms used may not have been sufficiently sensitive to detect such effects.

Li et al. [17] performed a two-step Mendelian randomization mediation analysis to investigate the associations between micronutrients, brain structure and function, and neurodevelopmental disorders through imaging-derived phenotypes (IDPs). The study integrated genome-wide association study (GWAS) datasets with 3935 brain IDPs derived from the UK Biobank neuroimaging dataset, including up to 33,224 individuals. The analyses examined potential causal relationships between genetically predicted micronutrient concentrations and neurodevelopmental disorders, including ADHD, autism spectrum disorder, and Tourette syndrome. Among the examined micronutrients, increased blood erythrocyte zinc concentration was associated with an 8% reduced risk of ADHD. Additional findings suggested protective associations involving magnesium and vitamin B12 for other neurodevelopmental conditions. Mediation analyses indicated that the observed associations were partly mediated through alterations in brain structure, neural activity, and functional connectivity. The identified imaging-derived phenotypes involved cortical thickness, cortical surface area, subcortical brain volumes, white matter microstructure, and resting-state functional connectivity measures. In relation to ADHD specifically, zinc-associated reductions in ADHD risk were mediated by imaging-derived phenotypes involving middle frontal, superior temporal, fusiform, and left caudate regions implicated in attentional regulation and executive functioning. These findings provided genetically informed evidence supporting a potential causal association linking micronutrient status, brain structure and function, and neurodevelopmental outcomes, although the mediation pathways remain inferential and require further validation.

**Figure 3:**
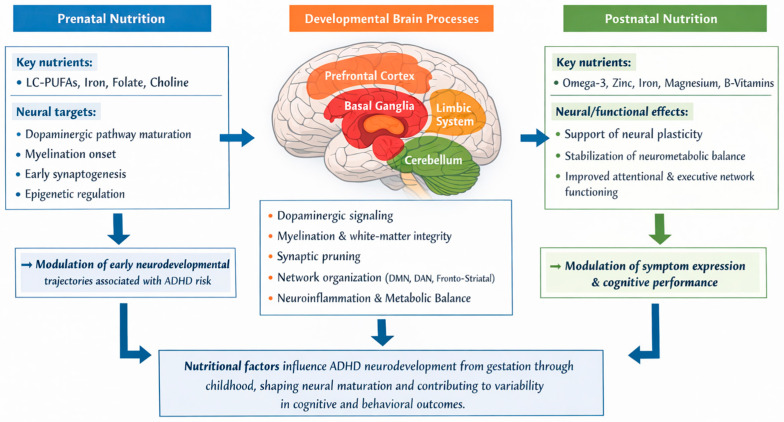
Nutritional influences on ADHD neurodevelopment. Prenatal and postnatal nutrients modulate key brain regions and networks—prefrontal cortex, basal ganglia, limbic system, cerebellum—affecting dopaminergic signaling, synaptic plasticity, and executive-attentional functions across development.

Overall, the included studies suggested that nutritional factors are associated with neurodevelopmental trajectories relevant to ADHD through relationships with structural brain development, functional activation, neurometabolic regulation, and large-scale network connectivity. Despite substantial heterogeneity in study design, neuroimaging methodology, nutritional exposures, and analytical approaches, converging findings implicated frontal, temporal, parietal, subcortical, and white matter systems involved in attentional control, executive functioning, inhibitory regulation, and cognitive processing. Prenatal nutritional exposures were associated with structural brain development patterns, whereas postnatal nutritional interventions showed preliminary associations with neurometabolic profiles and functional connectivity measures. However, several findings, particularly those related to micronutrient supplementation and exploratory connectivity analyses, were derived from secondary or exploratory analyses in relatively small samples and therefore require cautious interpretation. Collectively, the available evidence supports the biological plausibility that nutritional factors may modulate neural systems implicated in ADHD, while also highlighting the need for larger longitudinal studies, standardized neuroimaging methodologies, and adequately powered intervention trials to clarify causal mechanisms and clinical relevance. The main results are summarized in Figure 3.

## 4. Discussion

Across recent years, increasing attention has been directed toward the role of nutrition in the neurodevelopmental mechanisms underlying ADHD. The role of nutrition in neurodevelopment and in the emergence of ADHD has been investigated, yet the way in which brain structure and function may change within this context remains poorly understood. Specific micronutrients appear to have protective, genetically mediated causal roles in neurodevelopmental disorders, with their effects partly operating through modifications in brain architecture, neural activity, and connectivity patterns [17,18,19,20]. The influence of nutrition on ADHD is not limited to the prenatal period, and growing evidence shows that nutrients can continue to exert protective and therapeutic effects throughout childhood and adolescence. After birth, the brain remains in a prolonged phase of maturation marked by synaptic pruning, myelination, and the refinement of large-scale networks such as the default mode, dorsal attention, and fronto-striatal circuits [16,21]. These processes are metabolically demanding and remain sensitive to the availability of key nutrients. Omega-3 long-chain polyunsaturated fatty acids, for example, support membrane fluidity, neurotransmission, and anti-inflammatory balance, and supplementation during childhood has been associated with improvements in attention, executive functioning, and emotional regulation [1]. The studies included in this review suggest that nutritional factors may influence brain maturation and neurobiological processes associated with attentional and executive functioning. However, the currently available neuroimaging evidence remains limited and heterogeneous, preventing definitive conclusions regarding causality or clinical applicability.

The findings identified in this review support the hypothesis that specific nutritional factors may contribute to structural, functional, and metabolic brain characteristics associated with ADHD. Van Rooij et al. [12] reported that prenatal vitamin D supplementation, multivitamin use, folic acid supplementation, and overall diet quality were associated with specific structural brain volume patterns related to ADHD and ASD traits, suggesting that early nutritional exposure may influence neurodevelopmental trajectories relevant to attentional regulation and cognitive maturation. These findings support the hypothesis that prenatal nutrient availability may play an important role during critical periods of fetal brain development and cortical organization.

Borlase et al. [15] demonstrated that micronutrient supplementation was associated with reduced glutamate concentrations in the prefrontal cortex, increased gray matter volume in the anterior thalamus, increased white matter in the fornix, and improved resting-state network integrity. These findings suggest that micronutrients may influence neurometabolic regulation and connectivity within neural circuits involved in executive functioning and attentional control. Similarly, Bos et al. [16] observed reductions in inattentive symptoms following EPA/DHA supplementation in boys with and without ADHD, although no significant changes were detected in fMRI measures of brain activity. This discrepancy between clinical improvement and imaging findings may indicate that nutritional interventions can produce subtle neurofunctional effects that are not always detectable through current neuroimaging methodologies.

The reviewed evidence also highlights the possible protective role of micronutrient status in ADHD vulnerability. Using a two-step Mendelian randomization approach, Li et al. [17] reported that increased erythrocyte zinc concentration was associated with a reduced risk of ADHD, supporting the hypothesis that micronutrients may exert protective neurodevelopmental effects through mechanisms involving brain structure and function. Although these findings strengthen the biological plausibility of nutritional influences in ADHD, they remain indirect and require confirmation through longitudinal and interventional neuroimaging studies.

Taken together, the included studies suggest that nutrition may influence ADHD-related neurodevelopment through multiple and partially overlapping pathways, including prenatal brain maturation, neurometabolic regulation, white matter organization, and functional connectivity. Nevertheless, substantial methodological heterogeneity remains across studies, particularly regarding nutritional assessment methods, supplementation protocols, neuroimaging modalities, and outcome measures. The limited number of randomized neuroimaging studies, relatively small and often non-representative samples, and lack of long-term follow-up further restrict the interpretation and reproducibility of current findings. Several limitations should therefore be considered. First, the evidence base remains quantitatively limited, with only a small number of studies meeting inclusion criteria. Second, the included studies differed substantially in participant characteristics, imaging techniques, and nutritional interventions, limiting comparability across findings. Third, potentially relevant confounding factors—including socioeconomic status, medication use, baseline dietary quality, and psychiatric comorbidities—were not consistently controlled. Finally, most available studies cannot determine whether observed neurobiological differences represent causal mechanisms, compensatory adaptations, or secondary correlates of ADHD symptomatology. Future research should prioritize longitudinal and multimodal approaches integrating nutritional assessment, neuroimaging biomarkers, metabolic measures, and developmental outcomes. Larger randomized controlled trials will be necessary to determine whether targeted nutritional interventions can produce reproducible and clinically meaningful neurobiological changes in ADHD populations. Overall, current findings support nutrition as a biologically plausible but still incompletely understood factor within the broader neurodevelopmental framework of ADHD.

## 5. Conclusions

The findings reviewed converge on the idea that nutrition represents a potentially important biological influence on the neurodevelopmental processes implicated in ADHD. Available evidence suggests that both prenatal nutrient exposure and postnatal nutritional status may contribute to brain maturation, neurometabolic regulation, and the organization of neural networks involved in attention and executive functioning. In particular, prenatal vitamin D exposure, omega-3 fatty acids, and micronutrients such as zinc appear to be associated with structural or functional brain characteristics relevant to ADHD [12,15,16,17].

Although current evidence remains limited and heterogeneous, the reviewed studies support the hypothesis that nutrition may interact with neurodevelopmental mechanisms contributing to ADHD symptom expression. However, the available neuroimaging findings are still insufficient to establish definitive causal relationships or standardized nutritional interventions. Overall, nutrition should be considered a biologically plausible and potentially modifiable factor within the broader developmental context of ADHD. Further longitudinal and multidisciplinary research integrating nutritional, neuroimaging, genetic, and developmental data will be necessary to clarify the mechanisms linking diet, brain development, and ADHD, and to determine the potential clinical relevance of nutritional approaches in prevention and treatment strategies.

## Figures and Tables

**Table 1:** Risk of Bias Assessment (RoB 2.0)—Randomized Controlled Trials.

Study	Randomization Process	Deviations from Intended Interventions	Missing Outcome Data	Measurement of Outcomes	Selection of Reported Results	OverAll Risk of Bias
Borlase et al., 2020 [15]	Low—randomization adequately described and implemented	Low—no major deviations from the intended micronutrient/placebo intervention	Some concerns—minor imbalance in drop-outs	Low—standardized resting-state fMRI and MRS protocols	Low—outcomes consistent with preregistered protocol	Low
Bos et al., 2015 [16]	Some concerns—randomization described but with limited procedural detail	Low—good adherence to omega-3 supplementation	Low—outcome data largely complete	Low—validated neuropsychological and neuroimaging measures	Some concerns—not all outcomes explicitly prespecified	Some concerns

**Table 2:** Risk of Bias Assessment—Non-Randomized Studies (ROBINS-I).

Study	Design	ROBINS-I Assessment	Notes
van Rooij et al., 2025 [12]	Prospective cohort (Generation R)	Moderate risk—confounding inherent to observational prenatal exposure studies	Large population-based cohort; no causal inference possible
Li et al., 2025 [17]	Two-sample Mendelian Randomization	Low–Moderate risk—validity of genetic instruments varies across exposures	Genetic causal inference; not an interventional design

**Table 3:** Newcastle–Ottawa Scale Table.

Study	Selection (0–4)	Comparability (0–2)	Outcome/Exposure (0–3)	Total NOS Score (0–9)	Quality
van Rooij et al., 2025 [12]	4	1	2	7/9	Good
Li et al., 2025 [17]	3	1	2	6/9	Moderate

**Table 4:** JBI Critical Appraisal Checklist Table.

Study	Clear Inclusion Criteria	Valid/Reliable Exposure Measurement	Valid/Reliable Outcome Measurement	Identification of Confounders	Strategies to Deal with Confounders	Appropriate Statistical Analysis	Total Score (0–8)	Quality
van Rooij et al., 2025 [12]	Yes	Yes	Yes	Yes	Yes	Yes	6/8	Moderate–High

**Table 5:** Summary of all included articles, detailing study design, nutritional interventions, and the primary neurodevelopmental outcomes reported in children and adolescents with ADHD. Abbreviations: MRI, magnetic resonance imaging; ADHD, Attention-Deficit/Hyperactivity Disorder; EPA/DHA, eicosapentaenoic acid/docosahexaenoic acid; HC, Healthy control.

Authors	Year	Diagnosis	Study Sample (N)	Age (Mean)	Sex at Birth (% Female)	Type of Imaging	Outcome Measures	Main Results
[12]	2025	ADHD	3737	10.12	45%	MRI	volume childhood brain components	vitamin D and diet quality are associated with larger-volume brain components
[15]	2020	ADHD	27	10.75	0%	MRI; rs-fMRI	changes in neurometabolite levels through treatment with micronutrients	decreased glutamate in the prefrontal cortex; increased grey matter in the anterior thalamus; increased white matter in the fornix and improved network integrity
[16]	2015	ADHD	40 ADHD; 39 HC	ADHD: 10.3; HC:10.9	0%	fMRI	effects of dietary omega-3 fatty acid supplementation on ADHD symptoms and cognitive control	no effect of EPA/DHA supplementation on fMRI measures of brain activity, but a reduction of symptoms.
[17]	2025	ADHD	33,224	N/A	N/A	MRI	protective effects of micronutrients on neurodevelopmental disorders	increase in blood erythrocyte zinc concentration was associated with an 8% reduced risk of ADHD

## Data Availability

The original contributions presented in this study are included in the article and Supplementary Materials. Further inquiries can be directed to the corresponding author.

## Supplementary Materials

The following supporting information can be downloaded at: https://www.mdpi.com/article/10.3390/neurolint18060107/s1, Supplementary File: PRISMA checklist.
