# Supplementary material for: Nutritional Influences on the Brain in ADHD: Evidence from Neuroimaging Studies
Source: Neurol Int. 2026 May 29;18(6):107. doi: 10.3390/neurolint18060107 (PMC13305429; doi:10.3390/neurolint18060107)
Supplement: Supplementary file 1 [file neurolint-18-00107-s001.zip › neurolint-4240665-supplementary.pdf]

Section	Item	Checklist Item	Location in Manuscript (page)
**TITLE**	1	Identify the report as a systematic review.	1
**ABSTRACT**	2	Provide a structured summary including background, objectives, methods, results, and conclusions.	1
**INTRODUCTION**	3	Describe the rationale for the review.	2
	4	Provide an explicit statement of the objectives or questions addressed.	2
**METHODS**	5	Specify inclusion and exclusion criteria.	3
	6	Describe all information sources (databases, registers, etc.) and search dates.	3-4
	7	Present the full search strategies for all databases.	3-4
	8	Describe the selection process (screening, eligibility, reviewers).	3-4
	9	Describe the data collection process.	___3-4
	10	List and define all variables for which data were sought.	3-4
	11	Describe methods used to assess risk of bias in included studies.	4-5
**RESULTS**	16	Describe the results of the study selection process and include the PRISMA flow diagram.	4-6
	17	Present characteristics of included studies.	4-8
	18	Present assessments of risk of bias for each included study.	5
	19	Present results of individual studies.	_6-9__
	20	Present results of syntheses (narrative or quantitative).	6-9
**DISCUSSION**	23	Provide a general interpretation of the results.	10-11
	24	Discuss limitations of the evidence and of the review process.	11
	25	Provide implications for practice, policy, and future research.	11
**OTHER INFORMATION**	26	Indicate if a protocol exists and where it can be accessed (e.g., PROSPERO).	3
	27	Describe sources of financial or non-financial support.	12
	28	Declare competing interests.	12
